# Dopaminergic Neurons in Zona Incerta Drives Appetitive Self‐Grooming

**DOI:** 10.1002/advs.202308974

**Published:** 2024-08-05

**Authors:** Zhiying Jiang, Michelle He, Claire Young, Jing Cai, Yuanzhong Xu, Yanyan Jiang, Hongli Li, Maojie Yang, Qingchun Tong

**Affiliations:** ^1^ The Brown Foundation Institute of Molecular Medicine for the Prevention of Human Diseases The University of Texas Health Science Center at Houston Houston TX 77030 USA; ^2^ Summer Undergraduate Research Program The University of Texas Health Science Center at Houston Houston TX 77030 USA; ^3^ Sargent College of Health and Rehabilitation Sciences Boston University Boston MA 02215 USA; ^4^ MD Anderson Cancer Center & UTHealth Graduate School for Biomedical Sciences University of Texas Health Science at Houston Houston TX 77030 USA

**Keywords:** A13, dopamine, PAG, self‐grooming, zona incerta

## Abstract

Dopaminergic (DA) neurons are known to play a key role in controlling behaviors. While DA neurons in other brain regions are extensively characterized, those in zona incerta (ZI^TH^ or A13) receive much less attention and their function remains to be defined. Here it is shown that optogenetic stimulation of these neurons elicited intensive self‐grooming behaviors and promoted place preference, which can be enhanced by training but cannot be converted into contextual memory. Interestingly, the same stimulation increased DA release to periaqueductal grey (PAG) neurons and local PAG antagonism of DA action reduced the elicited self‐grooming. In addition, A13 neurons increased their activity in response to various external stimuli and during natural self‐grooming episodes. Finally, monosynaptic retrograde tracing showed that the paraventricular hypothalamus represents one of the major upstream brain regions to A13 neurons. Taken together, these results reveal that A13 neurons are one of the brain sites that promote appetitive self‐grooming involving DA release to the PAG.

## Graphical abstract

Dopaminergic neurons in the zona incerta (A13) integrate sensory information and drive self‐grooming and place preference by releasing dopamine to the periaqueductal gray (PAG). The dependency of place preference on self‐grooming suggests that self‐grooming is appetitive. These findings uncover a novel role for A13 neurons in regulating appetitive behaviors, offering exciting insights into brain function.

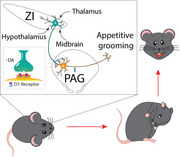

## 1 Introduction

Self‐grooming is an innate behavior that is important for hygiene maintenance, thermoregulation, de‐arousal, soothing after stress, and social attraction (for reviews, see,^[^ ^1^, ^2^ ^]^), and rodents spend a significant amount of their wake time grooming. Several brain regions have been identified as be involved in self‐grooming, including the orbitofrontal cortex (OFC), lateral septum (LS), ventral striatum (VS), islands of Calleja, lateral hypothalamus (LH), hypothalamic paraventricular nucleus (PVH), medial paralemniscal nucleus (MPL), substantia nigra (SNr) and ventral tegmental area (VTA).^[^ ^2^, ^3^, ^4^, ^5^, ^6^, ^7^, ^8^, ^9^, ^10^ ^]^ It is interesting to note that self‐grooming can be associated with positive or negative valence. While negative valence was observed to be associated with the self‐grooming behavior controlled by the islands of Calleja, LH, and PVH,^[^ ^5^, ^8^, ^10^ ^]^ positive valence was associated with the behavior controlled by the MPL.^[^ ^4^ ^]^ Surprisingly, both positive and negative emotions can be associated with the behavior related to the LS.^[^ ^5^, ^11^ ^]^ These observations collectively demonstrate that self‐grooming is controlled by multiple brain regions, serves as a common behavioral output, and is involved in a wide spectrum of emotional modalities.

Dopamine (DA) plays a vital role in encoding valence and directing behavior selections. DA neurons are found in both the midbrain and basal forebrain. DA neurons in the VTA project to the ventral striatum and are implicated in reward‐related behavior, while DA neurons in the SNr project to the dorsal striatum and play a predominant role in locomotor control,^[^ ^12^, ^13^, ^14^, ^15^, ^16^ ^]^ DA neurons in the arcuate nucleus are known to control endocrine function, and recent data also reveal a role for DA signaling in feeding regulation.^[^ ^17^ ^]^

Zona incerta (ZI) is a major subthalamic structure, representing a thin region located between the thalamus and hypothalamus. It can be divided into four cytoarchitechtural sectors: rostral, dorsal, ventral, and caudal^[^ ^18^, ^19^, ^20^ ^]^ without distinct boundaries or cellular specificity among these subregions. The ZI is heterogeneous, composed mainly of GABAergic neurons, with a small population of glutamatergic neurons.^[^ ^21^ ^]^ As its name reflects, the function of the ZI remained largely unknown until recently. In humans, deep brain stimulation of ZI improves motor function and mood in Parkinson's disease^[^ ^22^, ^23^ ^]^ however, it is unclear which subpopulation and projections mediate those effects. In rodents, whereas activation of glutamatergic neurons induces anxiety‐like behavior and jumping,^[^ ^24^ ^]^ different sectors and subpopulations of GABAergic neurons are involved in diverse behaviors: rostral ZI GABAergic neurons promote binge‐like eating^[^ ^25^ ^]^ and defensive behaviors,^[^ ^26^ ^]^ medial ZI GABAergic neurons drive predatory hunting,^[^ ^27^ ^]^ and ventral ZI GABAergic neurons promote sleep.^[^ ^28^ ^]^ Of note, ZI GABAergic neurons co‐express other biochemical markers such as parvalbumin, calbindin, tyrosine hydroxylase (TH, i.e., A13 neurons), somatostatin, calretinin, and serotonin.^[^ ^18^ ^]^ ZI parvalbumin neurons mediate flight behavior,^[^ ^29^ ^]^ fear memory,^[^ ^30^ ^]^ or modulate the sensation of itch;^[^ ^31^ ^]^ somatostatin neurons induce anxiety‐like behavior; and calretinin neurons play an anxiolytic role.^[^ ^24^ ^]^ However, little is known about the anatomy and function of ZI TH‐positive neurons. In this study, we aim to target TH‐positive dopaminergic neurons in the ZI area to examine their role in regulating behaviors.

## 2 Results

### 2.1 A13 TH+ Neurons Are Located in the Zona Incerta

**Figure 1:**
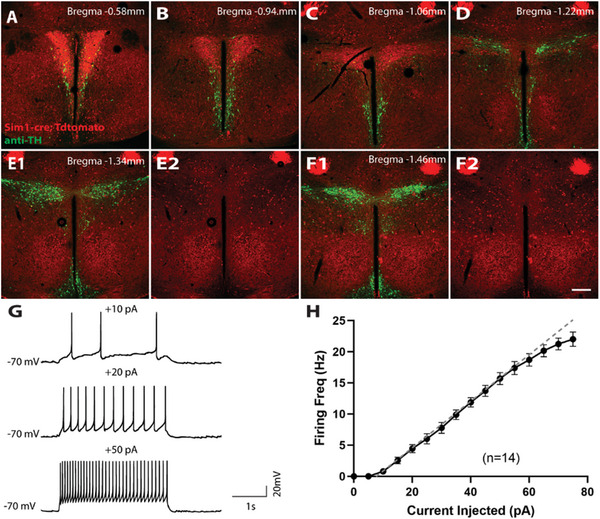
Distribution and electrical properties of ZI^TH^ neurons. A–F) Distribution of TH+ neurons (green) relative to PVH neurons (red). ZI^TH^ neurons are located posterior to the PVH region and are negative for PVH marker Sim1. Scale bar, 200 µm. G) firing of ZI^TH^ neurons upon varied current injections; H) *I–V* relationship of injected currents and average neuronal firing rates (*n* = 14 neurons).

A cluster of TH+ positive neurons, also called A13 neurons, is located in the ZI. Previous studies suggested that those neurons are part of the PVH and are implicated in energy balance regulation.^[^ ^32^ ^]^ The *Sim1* gene is a molecular marker for PVH neurons.^[^ ^33^ ^]^ Here, we used a S*im1‐cre::tdTomato* reporter mouse to mark the PVH and immunostained for TH. We found that the A13 TH+ neurons are posterior to the PVH, as the vast majority of them are negative for *Sim1* reporter expression (**Figure** **1**A–F). Those TH+ neurons are clustered in the rostral medial compartment of ZI. Consistent with previous observations, TH+ neurons in the ZI (ZI^TH^) are positive for vesicular GABA transporter (Vgat), a marker for GABAergic neurons (Figure S1A–D, Supporting Information). These data, together with previous studies showing that ZI^TH^ neurons are negative for dopamine‐𝛽‐hydroxylases, a marker for noradrenergic neurons, and positive for DOPA decarboxylase, and dopamine,^[^ ^21^, ^34^ ^]^ suggest that they are both GABAergic and dopaminergic. Interestingly, ZI^TH^ neurons are negative for dopamine transporter (DAT).^[^ ^34^, ^35^ ^]^ Therefore, ZI^TH^ neurons are not part of the PVH, and the *Th‐cre* but not *Dat‐Cre* mouse line can be used as a genetic tool to target those neurons. Approximately 70% of cre+ (GFP+) neurons are TH‐positive in adults (Figure S1E–H, Supporting Information). Injection of AAV into the ZI does not spread to the substantia nigra or ventral tegmental area (Figure S1M–P, Supporting Information).

To investigate the functions of ZI^TH^ neurons, we first characterized their intrinsic electrophysiological properties using whole‐cell patch‐clamp recordings in acutely prepared brain slices from *Th‐cre::L10A‐GFP* mice. ZI^TH^ neurons showed a resting membrane potential of −51.6 ± 1.9 mV (*n* = 13), and an input resistance of 1.04 ± 0.09 GΩ (*n* = 14). Half (7/13) of these neurons displayed spontaneous firing, with an average firing rate of 3.4 ± 0.9 Hz. Upon current injection, ZI^TH^ neurons fired action potentials reliably up to 20 Hz (Figure 1G,H). Therefore, we used an optogenetics protocol with stimulation frequencies up to 20 Hz to activate ZI^TH^ neurons for our following optogenetics experiments.

### 2.2 ZI^TH^ Neurons Bidirectionally Regulate Grooming Behavior

**Figure 2:**
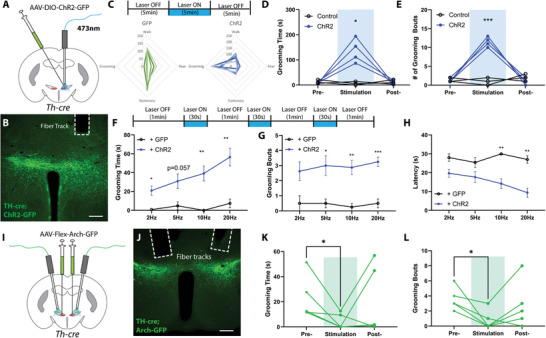
ZI^TH^ neurons bidirectionally regulate self‐grooming. A) Experimental diagram of optogenetic activation of ZI^TH^ neurons; B) a post‐hoc image illustrating the expression of AAVs and the location of optical fiber (scale bar, 200 µm); C) schematic diagram of optogenetic stimulation strategy, and summary radar plot of behaviors during laser exposure (control: *N* = 6 mice, ChR2: *N* = 7 mice); D,E) total grooming time and the number of grooming bouts in 5 min before, during, and after optogenetic stimulation of ZI^TH^ neurons (control, *N* = 3 mice, and ChR2: *N* = 4 mice, *p* = 0.032 and *p* = 0.0002, repeated measurements two‐way ANOVA). F–H) experimental strategy, a total grooming time, the number of grooming bouts, and latencies in the 90s induced by varying stimulation frequencies (*N* = 3 from control, and *N* = 4 from ChR2 mice). I) experimental diagram of optogenetic inhibition of ZI^TH^ neurons; J) a post‐hoc image illustrating the expression of AAVs and the location of optical fiber (scale bar, 200 µm); K,L) total grooming time and the number of grooming bouts in 5 min before, during, and after optogenetic inhibition of ZI^TH^ neurons (*N* = 5 mice, *p* = 0.046 and *p* = 0.031, paired *t*‐test). *p < 0.05, ***p < 0.001.

ZI neurons regulate diverse behaviors including ingestion, sensory processing, pain, and sleep.^[^ ^27^, ^28^, ^31^, ^36^ ^]^ To identify the function of ZI^TH^ neurons, we used an optogenetics approach. We injected Th‐cre mice with pAAV‐EF1a‐DIO‐hChR2(H134R)‐EYFP (ChR2) or AAVDJ8‐DIO‐GFP (Control) into the ZI and implanted an optic fiber 200 µm above the injection sites (**Figure** **2**A,B). Upon laser activation, mice exhibited a robust self‐grooming behavioral response (Figure 2C), as demonstrated by increased grooming bouts and duration (Figure 2D,E). In addition, the intensity of the elicited grooming behavior was stimulation dose‐dependent: higher stimulation frequencies induced longer grooming duration, and shorter latency, with no significant change in the number of grooming bouts (Figure 2F). In control mice, we didn't observe stimulation‐induced grooming (Figure 2C–H). In addition, the stimulation‐induced grooming was confined only to auto‐grooming, and mice didn't groom others (allogrooming) when littermates were present during stimulation (Video S1, Supporting Information). To assess the necessity of ZI^TH^ neurons for spontaneous self‐grooming, we expressed archaerhodopsin in the ZI (Figure 2I–J). Bilateral inhibition of ZI^TH^ neurons reduced both grooming time and frequency (Figure 2K–L), indicating their critical role in spontaneous grooming.

Rodent grooming behavior follows a highly stereotyped pattern, known as a syntactic chain, which can be divided into four stages: nose stroke (phase I), face stroke (phase II), head grooming (phase III), and body licking (phase IV). Grooming patterns are sensitive to the internal states of animals.^[^ ^1^ ^]^ Therefore, we further analyzed the microstructure of the grooming behavior induced by ZI^TH^ neuron activation.^[^ ^37^ ^]^ Interestingly, we found that the majority (67%) of the elicited grooming behavior proceeded to phase IV body‐licking, suggesting that the self‐grooming behavior elicited by activation of ZI^TH^ neurons follows a stereotyped cephalocaudal pattern. This cephalocaudal pattern of grooming is distinct from the repetitive grooming behaviors observed with other brain regions including the LH,^[^ ^10^ ^]^ ventral striatal islands of Calleja, and the PVH,^[^ ^7^, ^9^ ^]^ which involve‐ incorrect grooming transitions, interrupted grooming bouts, and is associated with stress and negative valence.

### 2.3 ZI^TH^ Neurons Encode Positive Valence

**Figure 3:**
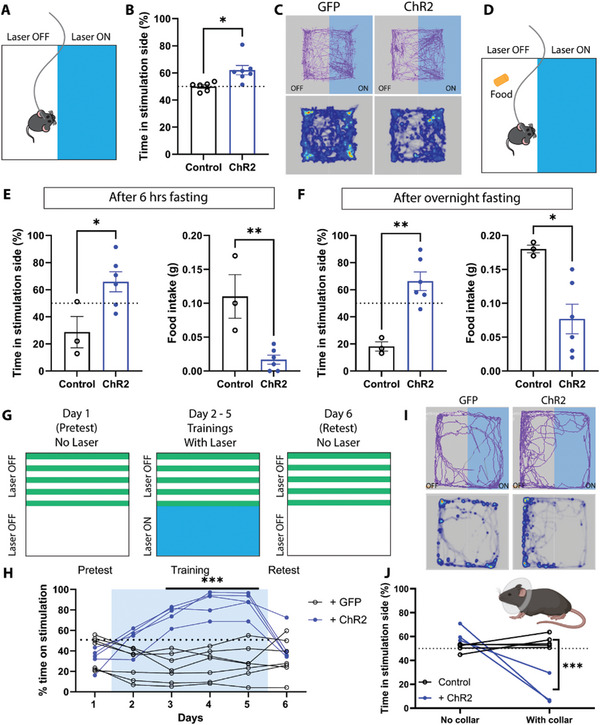
Optogenetic activation of ZI^TH^ neurons induces real‐time place preference (RTPP). A) Schematic diagram of real‐time place preference strategy; B) histogram of RTPP (control: *N* = 6 mice, ChR2: *N* = 7 mice, *p* = 0.011, two‐tailed unpaired Student's *t*‐test); C) representative animal trace tracks (top) and heatmap of time spent (bottom) in the arena from a GFP‐injected animal (left panels), and a ChR2‐injected animal (right panels); D) diagram of the experimental setup for RTPP paired with food; E) summary histograms of time spent on the stimulation side and the amount of food consumed during tests after 6 h food deprivation (control *N* = 7 mice, ChR2: *N* = 9 mice, *p* = 0.0005 and *p* = 0.018, respectively, two‐tailed unpaired Student's *t*‐test); F) summary histograms of time spent on the stimulation side and the amount of food consumed during tests after overnight food deprivation (control *N* = 6 mice, ChR2: *N* = 9 mice, *p* < 0.0001 and *p* = 0.0005, respectively, two‐tailed unpaired Student's *t*‐test); G) illustrations of the experimental setup for conditioned place preference (CPP); H) summary of time spent on the stimulation side during CPP tests (control *N* = 7 mice, ChR2: *N* = 5 mice, *p* < 0.001 on days 3–5, repeated measurements two‐way ANOVA). I) representative animal trace tracks (top) and heatmap of time spent (bottom) in the arena from a GFP‐injected animal (left panels), and a ChR2‐injected animal (right panels) with collar to prevent grooming; J) summary histogram of time spent on the stimulation side without and with Elizabethan collar (control *N* = 5 mice, ChR2: *N* = 3 mice, no collar *p* = 0.049, with collar *p* < 0.0001, repeated measurements two‐way ANOVA). Each circle represents an individual mouse. *p < 0.05; **p < 0.01.

Activation of ZI GABAergic neurons drives preference^[^ ^25^, ^27^ ^]^ or negative valence.^[^ ^24^ ^]^ To determine whether ZI^TH^ neurons encode either positive or negative valence, we ran a real‐time place preference (RTPP) test (**Figure** **3**A). We found that optogenetic activation of ZI^TH^ neurons elicited positive valence: mice preferred to stay on the side paired with optogenetic stimulation (Figure 3B,C). Next, to evaluate how strong the preference was, we paired the unstimulated side with food and ran an RTPP test with mice after 6 h of overnight fasting, which motivates food foraging behaviors (Figure 3D). Interestingly, even with increased food motivation after food deprivation, TH‐ChR2 mice still preferred to stay longer on the stimulated side (Figure 3E,F), which resulted in consuming less food (Figure 3E,F), suggesting that optogenetic activation of ZI^TH^ neurons promoted an appetitive drive that is even stronger than the motivation to eat food in a hungry state.

Some ZI^TH^ neurons are involved in memory formation and recall,^[^ ^30^, ^38^ ^]^ so we asked whether the appetitive drive could be transformed into memory. We did a conditioned place preference (CPP) test (Figure 3G). Mice did show an increased preference for the stimulated side during conditioning with a repeated stimulation protocol over several days (Figure 3H). On the test day when the stimulation was omitted, no preference was observed despite the strong preference observed during training (Figure 3H). In contrast, when laser stimulation was restored the following day, place preference was comparable to that seen during training (Figure S2, Supporting Information). To determine if place preference and grooming are related or independent, we trained mice to wear Elizabethan collars to prevent grooming (Figure 3J insert). Interestingly, when grooming was prevented, real‐time preference was blocked but instead real‐time aversion was observed (Figure 3I,J), suggesting that activation of ZI^TH^ neurons‐induced preference depends on self‐grooming and that self‐grooming is appetitive. The observation that the preference elicited by ZI^TH^ neurons could be trained but could not be conditioned is consistent with the results showing that ZI^TH^ neurons don't project to the hippocampus (Figure S3C,D, Supporting Information). Furthermore, ZI^TH^ neuron‐induced preference is mediated through increased self‐grooming.

### 2.4 Dopamine Promotes Self‐Grooming through Activation of ZI^TH^ Neurons

**Figure 4:**
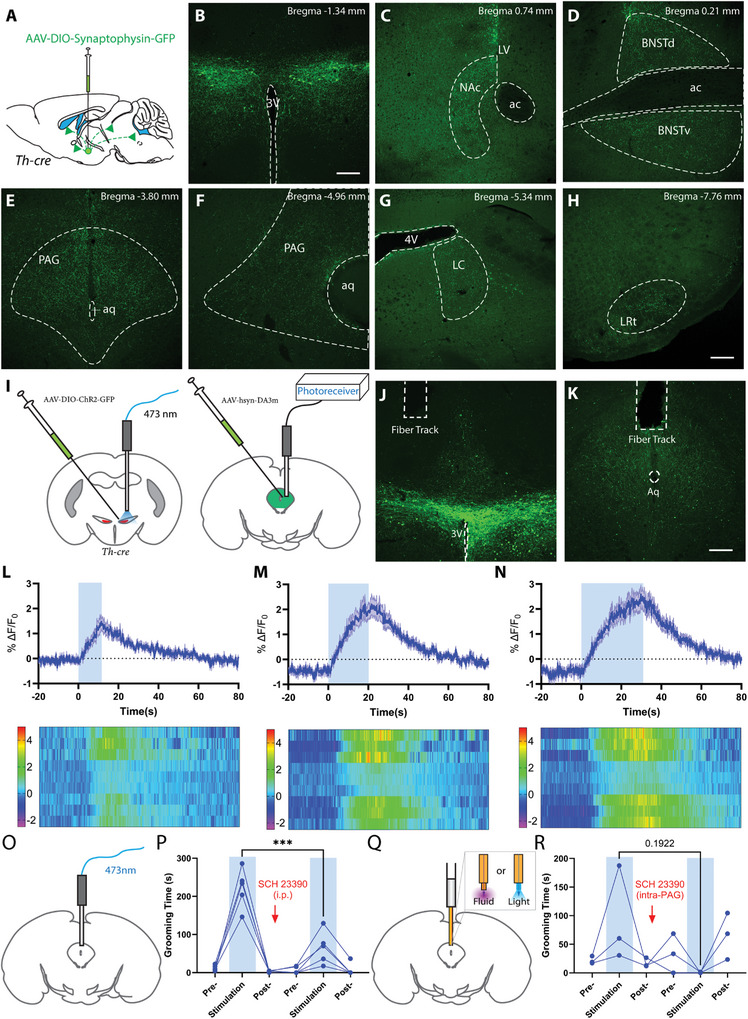
ZI^TH^ neurons release dopamine in the periaqueductal gray (PAG) and activate D1R in PAG to mediate self‐grooming. A) Schematic diagram for whole‐brain mapping of downstream targets of ZI^TH^ neurons; B) Expression of synaptophysin‐GFP at the injection sites in the ZI; C–H) Projections from ZI^TH^ neurons to nucleus accumbens shell, bed nucleus of the solitary terminus, PAG, locus coeruleus (LC), lateral reticular nucleus (LRt) (scale bar, 200 µm); I) Schematic diagram of strategy to measure DA release in the PAG with ZI^TH^ neurons activation; J,K) Images to show ChR2 and DA sensors expression, and fiber implantation; L–N) Ca2+‐dependent fluorescence changes before, during, and after optogenetic activation of ZI^TH^ neurons (*N* = 3 mice). O) Experimental setup to stimulate fiber terminals in the PAG; P) Time spent in grooming before, during, and after laser stimulation before and 15 min after intraperitoneal injection of D1R antagonist SCH23390 (*N* = 5 mice, *p* <0.001, repeated measurements one‐way ANOVA); O) Experimental setup to locally infuse drugs and stimulate fiber terminals in the PAG via an optofluidic cannula; P) Time spent in grooming before, during, and after laser stimulation before and 15 min after intra‐PAG injection of D1R antagonist SCH23390 (N = 3 mice, p = 0.192, repeated measurements one‐way ANOVA); ***p < 0.001.

To identify which downstream brain regions mediated the effects of DA release from ZI^TH^ neurons, we mapped the downstream targets of ZI^TH^ neurons. We injected AAVDJ8‐EF1a‐DIO‐synaptophysin‐GFP into the ZI of *Th‐cre* mice and examined the expression pattern of GFP (**Figure** **4**A,B), as synaptophysin‐GFP preferentially localizes in presynaptic axon terminals. GFP+ puncta were detected in diverse brain regions, including the nucleus accumbens shell (NAc, Figure 4C), bed nucleus of the stria terminalis (BNST, Figure 4D), periventricular gray, periaqueductal gray (PAG, Figure 4E,F), locus coeruleus (LC, Figure 4G), and lateral reticular nucleus (LRt, Figure 4H) (for a complete list, see Table S1, Supporting Information). However, ZI^TH^ neurons don't project to the dorsolateral striatum, or hippocampus (Figure S3A–D, Supporting Information). Notably, GFP+ terminals are found at multiple rostral‐caudal levels of the PAG. Since the PAG is known to be involved in complex motor functions, including grooming,^[^ ^39^ ^]^ we asked whether the ZI^TH^ neuron release of DA to the PAG is associated with self‐grooming behavior. To this end, we injected cre‐dependent AAV‐EF1a‐DIO‐hChR2(H134R)‐EYFP viral particles into the ZI of *Th‐cre* mice, a DA sensor (AAV‐hSyn‐DA3m)^[^ ^40^, ^41^ ^]^ into the PAG, and implanted optic fibers above both injection sites (Figure 4I–K). Upon laser stimulation to activate ZI^TH^ neurons, the PAG region demonstrated increased DA levels and self‐grooming behaviors, suggesting that activation of ZI^TH^ neurons releases DA to the PAG (Figures 4L–N). To identify whether DA release from ZI^TH^ neurons mediates grooming, we injected mice with a low dose of dopamine receptor D1 (D1R) antagonist SCH 23390 (0.1 mg kg^−1^, i.p.), as global knockout of D1R reduces syntactic self‐grooming, whereas knockout of D2R shows no effect.^[^ ^42^ ^]^ We found that a low dose of SCH diminished the activation of ZI^TH^ neurons‐induced self‐grooming (Figure 4Q,P). Intraperitoneal injection of a D1R antagonist blocks all D1R in the brain, including those outside the PAG that may be involved in self‐grooming. To specifically target D1R in the PAG, we expressed ChR2 in the ZI and implanted optofluid cannulas in the PAG. Local infusion of SCH23390 also blocked grooming induced by activation of ZI^TH^ neuron fibers, indicating that ZI^TH^ neuron‐induced grooming is mediated by their projection to the PAG, the release of dopamine, and activation of D1R in the PAG.

ZI^TH^ neurons send projections to other brain regions involved in grooming, such as the NAc, in which abundant dopamine receptors are expressed.^[^ ^4^ ^]^ Therefore, we also tried fiber implantation in the NAc. While we detected strong endogenous DA release in the NAc, the DA signals were not correlated with self‐grooming behaviors elicited by laser stimulation (Figure S3E,F, Supporting Information), suggesting that it is unlikely that DA action in the NAc mediates ZI^TH^ neurons‐induced self‐grooming. This is consistent with previous observations that the ventral striatum (including the NAc) exhibits a decrease in DA signaling during spontaneous self‐grooming.^[^ ^3^ ^]^ Taken together, DA release contributes to the self‐grooming behavior elicited by ZI^TH^ neurons and the PAG is one of the downstream sites that mediate the effect.

### 2.5 ZI^TH^ Neurons Exhibit Increased Activity During Spontaneous Self‐Grooming

To ascertain the physiological relevance of the action of ZI^TH^ neurons on self‐grooming behaviors, we performed in vivo calcium imaging to monitor the activity of those neurons using fiber photometry during natural self‐grooming behavior. To this end, we injected *Th‐cre* or *Th‐cre;vgat‐flp* mice with AAV9‐hsyn1‐flex‐GCaMP6m‐EGFP or AAV8‐EF1A‐Coff/Fon‐GCaMP6f into the ZI, and implanted an optic fiber right above the injection site (Figure S4A,B, Supporting Information). ZI^TH^ neurons showed increased calcium signals during spontaneous self‐grooming (Figure S4C, Supporting Information), suggesting a role for ZI^TH^ neurons in promoting spontaneous self‐grooming.

**Figure 5:**
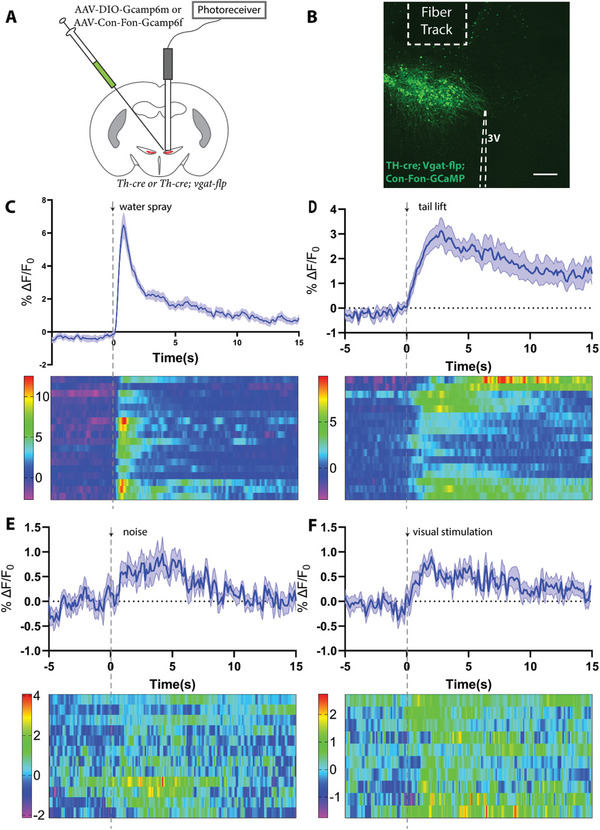
ZI^TH^ neurons are responsive to sensory inputs. A) Experimental diagram for the recording of calcium signals from ZI^TH^ neurons. B) Image to show the expression of GCaMP 6f and location of implanted fiber (scale bar, 200 µm). C–F) Time course and heatmap illustrating ZI^TH^ neurons calcium signals in response to stimuli: water spray (*N* = 7 mice, and *n* = 18 trials), tail lift (*N* = 5 mice, and *n* = 17 trials), noise (*N* = 4 mice, and *n* = 12 trials), and visual stimulation (*N* = 4 mice, *n* = 10 trials).

The connectivity of ZI neurons suggests that they have a role in sensorimotor integration.^[^ ^43^, ^44^ ^]^ Here, we found that the calcium signals in ZI^TH^ neurons also responded to various external stimuli, including water spray (Figure 4C), tail lift (Figure 4D), visual stimulation with a green laser (**Figure** **5**E), and high‐pitch noise from a dog whistle (Figure 5F), food presentation, and introduction of familiar or unfamiliar conspecifics (data not shown), suggesting that ZI^TH^ neurons can respond to a broad range of environmental cues. This is reminiscent of PVH corticotropin‐releasing hormone (CRH) neurons, which show increased neuronal activity in response to external stimuli, especially aversive stimuli.^[^ ^45^, ^46^, ^47^ ^]^ However, PVH CRH neurons are glutamatergic, and TH+ neurons are GABAergic, In addition, CRH neurons do not colocalize with ZI^TH^ neurons (Figure S5, Supporting Information). Therefore, these data confirm that ZI^TH^ neurons exhibit increased activity during spontaneous self‐grooming and respond to diverse environmental cues.

### 2.6 Mapping ZI TH+ Neurons Monosynaptic Inputs

**Figure 6:**
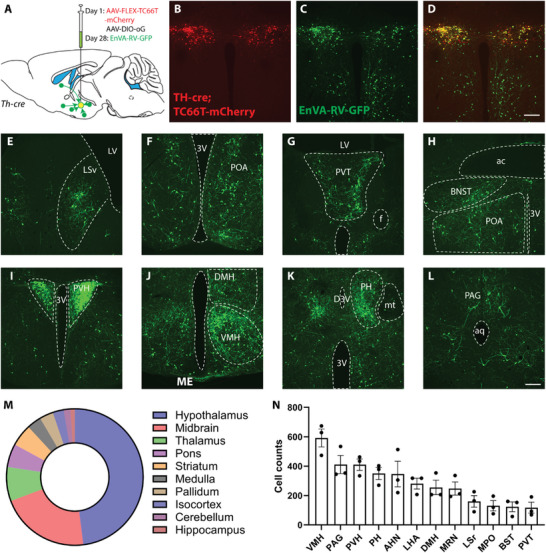
Whole‐brain mapping of monosynaptic inputs to ZI^TH^ neurons. A) Schematic experimental design for pseudotyped rabies virus‐assisted monosynaptic retrograde tracing; B–D) The selective expression of TC66T‐mCherry (red) in TH‐cre neurons within the ZI and rabies virus (green) infected neurons within and outside of ZI; D) Merged image showing the presence of starter neurons; E–L) Representative images of presynaptic neurons found in the ventral lateral septum (LSv), preoptic area (POA), anterior paraventricular nucleus of the thalamus (PVT), bed nucleus of solitary terminalis (BNST), paraventricular nucleus of the hypothalamus (PVH), dorsam and ventral medial hypothalamus (DMH, VMH), posterior hypothalamus (PH), and periaqueduct gray (PAG) (scale bars, 200 µm); M,N) Quantification of monosynaptic inputs to ZI^TH^ neurons, and top 12 upstream brains regions.

The ZI receives inputs from multiple sensory pathways, and each division of the ZI has a specific connection pattern.^[^ ^48^ ^]^ To identify the monosynaptic upstream neurons, *Th‐cre* mice were injected with a mixture of AAVDJ8‐CAG‐FLEX‐TC66T‐mCherry, and AAVDJ8‐EF1A‐DIO‐oG‐WPRE‐hGH, followed by another injection of EnVA‐RV‐GFP 4 weeks later (**Figure** **6**A–D). GFP‐positive neurons were identified in many brain regions, including the LS (Figure 6E), preoptic area (POA, Figure 6F), paraventricular nucleus of the thalamus (PVT, Figure 6G), the bed nucleus of the stria terminalis (BNST, Figure 6H), PVH (Figure 6I), ventromedial hypothalamic nucleus (VMH, Figure 6J), posterior hypothalamus (PH, Figure 6K), and PAG (Figure 6L) (for a complete list, see Table S2, Supporting Information), suggesting that ZI^TH^ neurons receive monosynaptic inputs from diverse brain regions (Figure 6M–N). The most robust inputs come from the VMH and PVH in the hypothalamus (Figure 6I–J).

## 3 Discussion

In this study, using optogenetics, fiber photometry, and circuit mapping, we demonstrated that activation of ZI^TH^ neurons drives appetitive emotions and induces intensive self‐grooming. In addition, we found that ZI^TH^ neurons exhibited increased neuronal activity during spontaneous self‐grooming, and were responsive to multiple sensory stimuli. Anatomically, these neurons have complex network connectivity, especially with the hypothalamus and midbrain neurons. Together, these data suggest that ZI^TH^ neurons serve as an important integrative node for diverse sensory stimuli and drive appetitive behaviors.

Distinct subpopulations of GABAergic neurons in the ZI exhibit functional heterogeneity, including binge‐like eating, predatory hunting, defensive behaviors, itch, sleep, etc. Here, we found that ZI^TH^ neurons are notable for eliciting intensive self‐grooming, a behavior distinct from other ZI neuron subtypes. Unlike A13 dopaminergic neurons projecting to the thalamic nucleus reunions involved in fear memory extinction recall,^[^ ^38^ ^]^ optogenetic activation of ZI^TH^ neurons inducing RTPP does not convert to contextual memory. Our results suggest a significant role of dopamine in the observed self‐grooming, highlighting it as a unique behavior associated with ZI^TH^ neuron activation. Thus, our study contributes to understanding the diverse behavioral effects mediated by ZI neurons.

Self‐grooming could be associated with positive or negative valence and the pattern of self‐grooming is highly sensitive to physiological and emotional states. Self‐grooming with interrupted bouts and incorrect transitions is often observed in stressed conditions,^[^ ^8^, ^9^ ^]^ and more rigid grooming patterns are found in rodent models of obsessive‐compulsive disorders (OCD), Tourette syndrome, and autism spectrum disorder (ASD). In contrast, spontaneous self‐grooming under non‐stressful conditions generally follows a stereotyped cephalocaudal sequence. Here, our study clearly demonstrates a positive valence associated with self‐grooming induced by ZI^TH^ neuron activation. Consistently, the self‐grooming behavior observed here also follows a stereotyped cephalocaudal sequence, suggesting that ZI^TH^‐induced self‐grooming is related to non‐stressful self‐grooming. In this regard, ZI^TH^‐induced grooming is similar to the grooming induced by MPL neurons reported previously.^[^ ^4^ ^]^ Intriguingly, MPL neurons increase c‐fos expression in response to stress^[^ ^4^ ^]^ and ZI^TH^ neurons also respond to various environmental cues including stressful stimuli, which invites the possibility that self‐grooming elicited by these neurons serves to reduce the negative feeling imposed by perceived stresses. This possibility is supported by our observations that prevention of grooming blocks activation of ZI^TH^ neurons‐induced preference, and it is further supported by evidence that ZI^TH^ neurons receive direct monosynaptic projections from a large number of PVH neurons. Since PVH neurons are known to respond to various environmental stressful cues and induce self‐grooming associated with negative valence, and the majority of PVH neurons are glutamatergic, the direct glutamatergic input from PVH to ZI^TH^ neurons may mediate the observed responses of ZI^TH^ neurons to environmental cues. It will be interesting to examine whether the self‐grooming behavior induced by PVH neurons is dependent on the function of ZI^TH^ neurons. The difference between associated negative valence by PVH neuron activation and positive valence by ZI^TH^ neuron activation may be mediated through differential downstream projections of these two groups of neurons via releasing glutamate or DA (potentially GABA) respectively. Further studies are warranted to examine this possibility.

Several signaling pathways are involved in self‐grooming, such as DA, serotonin, glutamate, and somatostatin.^[^ ^2^, ^3^, ^4^, ^7^, ^10^, ^49^ ^]^ DA signaling could bi‐directionally regulate self‐grooming, depending on projection sites and downstream dopamine receptor signaling.^[^ ^3^ ^]^ Here, our study provides another piece of evidence that dopamine is released from ZI neurons to activate D1R‐expressing neurons in the PAG to promote grooming, which is in line with previous findings that syntactic chain grooming is enhanced by DA signaling and D1R activation.^[^ ^50^ ^]^ Human studies suggest‐ that the ZI plays a role in improving motor and mood symptoms in patients with Parkinson's disease, however, the exact subtypes of neurons involved remain to be identified. Here, we found that ZI^TH^ neurons could both regulate syntactic grooming and encode positive valence, suggesting they may serve as a therapeutic target for the treatment of Parkinson's disease. ZI^TH^ neurons are both dopaminergic and GABAergic, expressing both Vmat2 and Vgat, and the function of GABA release from ZI^TH^ neurons remains to be answered. Although GABAergic neurotransmission is likely to decrease grooming behavior,^[^ ^51^ ^]^ whether it mediates the real‐time preference phenotype is unclear. Future studies with a focus on GABA release from ZI^TH^ neurons will be required to address this issue.

Previous studies suggest that ZI^TH^ neurons are sensitive to aversive stimuli including pain and itch.^[^ ^31^, ^36^, ^52^ ^]^ Here, our data support that ZI^TH^ neurons increase their neuronal activity in response to various sensory stimuli, including somatosensory, visual, auditory, and olfactory, suggesting that ZI^TH^ neurons are versatile in responding to various sensory inputs, and in this way may serve as an important integration center for mediating adaptive responses.^[^ ^53^ ^]^ Our findings contrast with those of Zhang et al., who recently demonstrated that ZI DA (ZI^TH^)neurons regulate food‐seeking behavior and contextual memory through projections to the PVT (Q. Ye, et al., 2023). While both studies highlighted the involvement of ZI^TH^ neurons in appetitive behaviors, our research specifically identified them with self‐grooming and DA‐mediated effects on the PAG, with no involvement in Pavlovian conditioning. In contrast, Zhang et al. showed that ZI^TH^ neuron activation enhanced both acquisition and expression of contextual food memory via operant conditioning. Differences in experimental conditions, such as task type (Pavlovian vs. instrumental conditioning), manipulation methods (optogenetic vs. chemogenetic activation), and environmental context (one chamber with the same flooring vs. three chamber with different floorings), might account for these divergent results. Additionally, our study observed increased grooming and decreased food intake with ZI^TH^ neuron activation, whereas Zhang et al. reported increased food intake. Together, these studies underscore the complex and context‐dependent roles of ZI^TH^ neurons in motivational and behavioral processes.

A limitation of our study is the exclusive use of male subjects. While our research provides valuable insights into the role of ZI^TH^ neurons in driving intensive self‐grooming, it is crucial to acknowledge that biological and behavioral responses may exhibit gender‐specific variations. Future studies are necessary to incorporate female subjects to comprehensively explore potential sex differences in the observed behaviors. Another limitation of our study lies in the interpretation of the conditioned place preference (CPP) results. While we observed that mice spent a longer time on the stimulated side with training, nonetheless, this behavior depends on the presence of a reward. The extended stay of mice on the stimulated side is driven by the anticipation of a reward rather than the association of the rewarding stimulus with the contextual environment.

## 4 Conclusion

Our study revealed that ZITH neurons are essential for appetitive self‐grooming via dopamine release to the periaqueductal gray. It highlights their role in sensory integration and spontaneous grooming behavior and offers a comprehensive mapping of their synaptic connectivity.

## 5 Experimental Section

### Animals

Adult mice aged 8–10 weeks were used in these experiments. The founders of the following transgenic mice were purchased from Jax Laboratory, and bred on a C57/BL6 background in house: *Th‐cre* (B6.Cg‐7630403G23Rik^Tg(Th‐cre)1d^/J, strain #:008601, RRID: IMSR_JAX:008601), L10A‐GFP (B6;129S4‐Gt(ROSA)26Sor^9(EGFP/Rpl10a)Amc^/J, strain # 024750, RRID: IMSR_JAX:024750), and Vgat‐flp (B6.Cg‐Slc32a1^1.1(flpo)Hze^/J, strain #:029591, RRID: IMSR_JAX:029591. All mouse procedures were approved by the University of Texas Health Science Center at Houston Institutional Animal Care and Use Committee.

### Stereotaxic Surgeries

Adult *Th‐cre* mice (8–10 weeks) were anesthetized with a mixture of ketamine (100 mg kg^−1^) and xylazine (10 mg kg^−1^), and placed on a stereotaxic frame. AAVs were delivered bilaterally to the ZI (100 nL per site at 50 nL min^−1^) using the following coordinates (distances from bregma): AP: −1.35 mm, ML: ± 0.40 mm, DV: −4.5 mm, with a Nanoinjector (Drummond Scientific). The injection needle was maintained in place for 5 min following injections to minimize virus spread up the needle track. The viruses used were pAAV‐EF1a‐DIO‐hChR2(H134R)‐EYFP‐WPRE‐HGHpA (2.1 × 10^13^ GC mL^−1^, Addgene 20298‐AAV5)

AAVDJ8‐DIO‐GFP (8.38 × 10^11^ GC mL^−1^, Baylor College of Medicine Viral Core), AAVDJ8‐EF1A‐DIO‐oG‐WPRE‐hGH (Salk Institute), AAVDJ8‐CAG‐FLEX‐TC66T‐mCherry (Baylor College of Medicine Viral Core), EnVA RV‐GFP (Salk Institute), AAV9‐hsyn1‐flex‐ GCaMP 6m‐EGFP (Addgene‐100838), AAV8‐EF1A‐Coff/Fon‐GCaMP6f (2.1 × 10^13^GC mL^−1^, Addgene 137124), AAV8‐Ef1a‐Con/Foff2.0‐GCaMP6f (2.6 × 10^13^ GC mL^−1^, Addgene 137123), AAV9‐hSyn‐DA3m (1.30 × 10^13^GC mL^−1^, WZ Biosciences), AAV9‐CAG‐FLEX‐Arch‐GFP (1.9 × 10^13^GC mL^−1^, Addgene‐22222). For optogenetics and fiber photometry studies, an optic fiber was implanted 200 µm (for optogenetics), and within 100 µm (for fiber photometry) right after AAV injections. The optic fibers were implanted at a 10° angle for the bilateral inhibition experiment. Mice were allowed to recover for at least 3 weeks before being used for experiments.

### Acute Brain Slices Preparation and Electrophysiological Recording

Electrophysiological experiments were conducted in acutely prepared hypothalamic slices, as previously described.^[^ ^54^, ^55^ ^]^ Briefly, adult *Th‐cre*; L10A‐GFP mice were deeply anesthetized with a mixture of ketamine/xylazine (intraperitoneally) and transcardially perfused with ice‐cold cutting solution containing the following (in mm): 75 sucrose, 73 NaCl, 26 NaHCO_3_, 2.5 KCl, 1.25 NaH_2_PO_4_, 15 glucose, 7 MgCl_2_, and 0.5 CaCl_2_, saturated with 95% O_2_/5% CO_2_. The brains were quickly removed from the skull and blocked, and the rostral face of the block was glued to the specimen plate of the buffer tray and then immersed in an ice‐cold cutting solution. Coronal slices (280 µm) containing the ZI were sectioned using a Leica VT1000S Vibratome and transferred to a holding chamber with artificial CSF (aCSF) containing the following (in mm): 123 NaCl, 26 NaHCO_3_, 2.5 KCl, 1.25 NaH_2_PO_4_, 10 glucose, 1.3 MgCl_2_, and 2.5 CaCl_2_, and saturated with 95% O_2_/5% CO_2_ at 31–33 °C for 30 min, then maintained at room temperature for at least 1 h to allow for recovery before any electrophysiological recordings.

Individual slices were transferred from the holding chamber to a recording chamber, where they were submerged and continuously perfused with oxygenated aCSF at ∼2 ml/min. TH neurons in the ZI were first located under epifluorescence illumination, and whole‐cell patch‐clamp recordings were performed on identified TH+ neurons under infrared‐differential interference contrast visualization at 30–32 °C on a fixed‐stage, upright microscope (model BX51WI, Olympus) equipped with a water‐immersion 40× objective. Pipettes with a resistance of 3–5 MΩ were pulled from borosilicate glass (outer diameter, 1.5 mm; inner diameter, 1.1 mm; Sutter Instruments) using a horizontal puller (P‐97, Sutter) and filled with an internal patch solution containing the following (in mm): 142 K‐gluconate, 10 HEPES, 1 EGTA, 2.5 MgCl_2_, 4 Mg‐ATP, 0.3 Na‐GTP, and 10 Na_2_‐phosphocreatine, adjusted to pH 7.25–7.35, osmolality 295–305 with KOH. The liquid junction potential was not corrected, and the series resistance (Rs) was bridge‐balanced.

### Immunohistochemistry (IHC) and Imaging

For histology studies, adult mice were deeply anesthetized with a ketamine (100 mg kg^−1^) and xylazine (10 mg kg^−1^) mixture, then transcardially perfused with saline followed by 10% neutral buffered formalin. Brains were removed, fixed in formalin overnight, equilibrated in 30% sucrose, sectioned (30 µm, coronal sections) on a frozen sliding microtome into four series, and stored in PBS with 0.1% sodium azide at 4 °C. Brain sections were rinsed with PBS and incubated with primary antibodies [goat anti‐GFP, 1:1000, catalog #600‐101‐21, Rockland (RRID:AB_218182); rabbit anti‐TH, 1:1000, catalog # AB112, Abcam (RRID:AB_297840)] in 2% normal donkey serum and 0.4% Triton X‐100 at 4 °C overnight. They were then incubated in secondary antibodies (all secondaries were obtained from Jackson Immuno‐Research: Alexa Fluor 488 donkey anti‐goat, catalog #705‐546‐147 (RRID:AB_2340430); Alexa Fluor 488 donkey anti‐rabbit, catalog #711‐546‐152(RRID:AB_2340619); Alexa Fluor 594 donkey anti‐rabbit, catalog # 711‐586‐152 (RRID: AB_2340622); 1:1000] at room temperature for 1 h. Sections were washed in PBS, mounted, and imaged on a confocal microscope (model TCS SP5, Leica).

### Real‐Time Place Preference (RTPP)

RTPP was run in a clear 45 × 45 × 50 cm^3^ chamber equipped with a camera mounted on the top of the chamber and an optical fiber patch cable attached to a commutator (PhenoTyper, Noldus). The chamber was divided into two equal zones: the light ON and the light OFF zones. Prior to the start of the experiments, mice were tethered to the patch cable, and placed in the OFF zone where no laser will be applied. Then, mice were allowed to explore freely in the chamber for 20 min, during which laser pulses (20 Hz, 10 ms) were delivered whenever mice entered the ON zone. The side paired with laser stimulation was counterbalanced between mice. Time spent in each zone was tracked and calculated by Ethiovision XT 15 software (Noldus), averaged from two trials run on opposite sides at least one week apart.

### Conditioned Place Preference

Conditioned place preferences were run in another Noldus Phenotyper chamber, with green tape on the outside of one half of the chamber to create a context environment. The taped side was assigned as the light OFF zone, due to the mice's innate preference to choose the taped side as it is slightly dimmer than the other side. Each day, mice were tethered to the patch cable and allowed to explore the chamber freely for 20 min. On day 1 (pre‐test) and day 6 (re‐test), no laser was applied. On days 2–5, blue laser pulses were delivered whenever mice entered the light ON zone. Time spent in each zone was tracked and calculated.

### Optogenetics

Mice were placed in the clear Noldus Phenotyper chamber, tethered to an optical patch cable. They were allowed to roam freely for 5 min, followed by 5 min laser stimulation (20 Hz, 10 ms), and then another 5 min without laser stimulation. For stimulation dose‐dependent responses, mice were given 30s stimulation at varied frequencies (2, 5, 10, and 20 Hz) for 3 trials each, with 1‐min intervals between trials.

### Behavior Quantification

Mice activities in the Noldus Phenotyper chamber were video‐recorded, and behavioral video analysis was conducted by an individual blinded to treatment groups using an open‐source event‐logging software BORIS.^[^ ^56^ ^]^ Walking is defined as an animal changing its location, rearing is defined as an animal temporarily standing on its hind limbs to investigate the environment, and grooming is defined as an animal licking or brushing its body with its front paws.

### Intra‐PAG Drug Infusion

To block dopamine transmission specifically in the PAG, mice were injected with AAVs in the zona incerta and implanted with optofluid guide cannulas (Doric Lenses, Canada) in the PAG. On the day of the experiment, guide cannula inserts were removed and optic fibers were inserted to establish a baseline response. Mice were then anesthetized with isoflurane, and the optic fibers were exchanged with injectors. Drugs (500 ng) were injected using a microinjection pump (Stoelting) at a rate of 100 nL min^−1^, and the injectors were left in place for 5 min to prevent backflow. Subsequently, optic fibers were reinserted, and mice were allowed to recover for an additional 10 min before returning to the behavioral arena.

### PTRV‐Mediated Monosynaptic Retrograde Tracing

To map upstream neurons that make monosynaptic inputs onto TH+ neurons in the ZI, a mixture of AAVs: AAVDJ‐CAG‐FLEX‐TC66T‐mCherry and AAVDJ‐EF1A‐DIO‐oG‐WPRE‐hGH were delivered to the ZI of the *Th‐cre* mice. Four weeks later, a second injection of pseudo rabies viruses EnVA RV‐GFP was delivered to the same location. Mice were perfused 10 days later.

For quantification of monosynaptic inputs, brain slices were registered to the Allen Mouse Common Coordinate Framework version 3 (CCF v3).^[^ ^57^ ^]^ Brightness and contrast were enhanced in images used for registration. Sectioning angles, AP coordinates, and linear transformations were applied using QuickNII.^[^ ^58^ ^]^ Nonlinear transformations for slice deformations were applied using VisuAlign v0.8.^[^ ^59^ ^]^ Brain slice images were segmented in Fiji^[^ ^60^ ^]^ (Schindelin et al., 2012) using a custom macro designed to identify neuronal soma and exclude fibers. A binary segmentation mask was generated for each brain slice and artifacts such as bubbles were manually removed. The segmentation mask and the registration output were combined in Nutil^[^ ^61^ ^]^ to calculate the number of neurons per brain region.

### Fiber Photometry

Mice were connected to the imaging patch cord (Doric Lenses, Canada) or the stimulating and imaging patch cords, and placed in a clean cage. Fluorescence signals were collected with an integrated fluorescence mini cube (Doric Lenses) and Doric Neuroscience Studio 5 or 6 software (Doric Lenses). For stimulated dopamine release experiments, a blue laser (473 nm, 20 Hz, 10 ms) was delivered through the stimulation cable driven by a Master‐8 pulse stimulator (AMPI, Israel).

For water spray, a mist of water was released from a sprayer bottle to the face of the mice; for tail lift, the tails of mice were grabbed by the experimenter, and the mice were suspended in the air for 10–15s; for visual stimulation, a green laser was pointed to the cage bedding in front of the mice; for auditory stimulation, a dog whistle was blown for 10–15s to create a high‐pitch noise.

### Fiber Photometry Data Analysis

Fiber photometry data were analyzed with customized code in Python following the previously described method.^[^ ^62^ ^]^ Briefly, first, data from signaling and isosbestic channels were extracted. Then, signals were smoothed using a Savitzky‐Golay filter to reduce high‐frequency noise. Next, signals from the isosbestic channel were normalized using an equation: scaled_control = a * control + b, where a polynomial function was used to find the relationship between the signal and control channel. Finally, ΔF/F was calculated as ΔF/F = (signal – scaled_control)/scaled_control. Data were downsampled to 10 Hz from 40–60 Hz by using the mean for visualization in GraphPad Prism.

### Statistical Analysis

Data are presented as the means ± SEM. All data were analyzed for statistical significance using ANOVA or a two‐tailed unpaired Student's *t*‐test with GraphPad Prism 9 and 10 software. A capital N refers to the number of animals used, while a small n represents the number of trials/neurons. Mice with missed viral injection or fiber implanted far from the target regions were excluded from the analysis.

## Conflict of Interest

The authors declare no conflict of interest.

## Author Contributions

Q.T. and Z.J. conceived and designed the experiment. Z.J. conducted the experiment. Z.J., M.H., and C.Y. analyzed and visualized the data. Z.J. and Q.T. wrote the manuscript. Z.J., J.C., C.Y., Y.J., Y.X., H. L., M.Y., and Q.T. reviewed and edited the manuscript.

## Supporting information

Supporting Information

Supplemental Video 1

## Data Availability

The data that support the findings of this study are available from the corresponding authors upon reasonable request.
