# Supplementary material for: Dopaminergic Neurons in Zona Incerta Drives Appetitive Self‐Grooming
Source: Adv Sci (Weinh). 2024 Aug 5;11(36):2308974. doi: 10.1002/advs.202308974 (PMC11422805; doi:10.1002/advs.202308974)
Supplement: Supplementary file 1 — Supporting Information [file ADVS-11-2308974-s001.pdf]

ADVANCED SCIENCE
Open Access

Supporting Information

for *Adv. Sci.*, DOI 10.1002/advs.202308974

Dopaminergic Neurons in Zona Incerta Drives Appetitive Self-Grooming

Zhiying Jiang, Michelle He, Claire Young, Jing Cai, Yuanzhong Xu, Yanyan Jiang, Hongli Li, Maojie Yang and Qingchun Tong**

Supporting Information

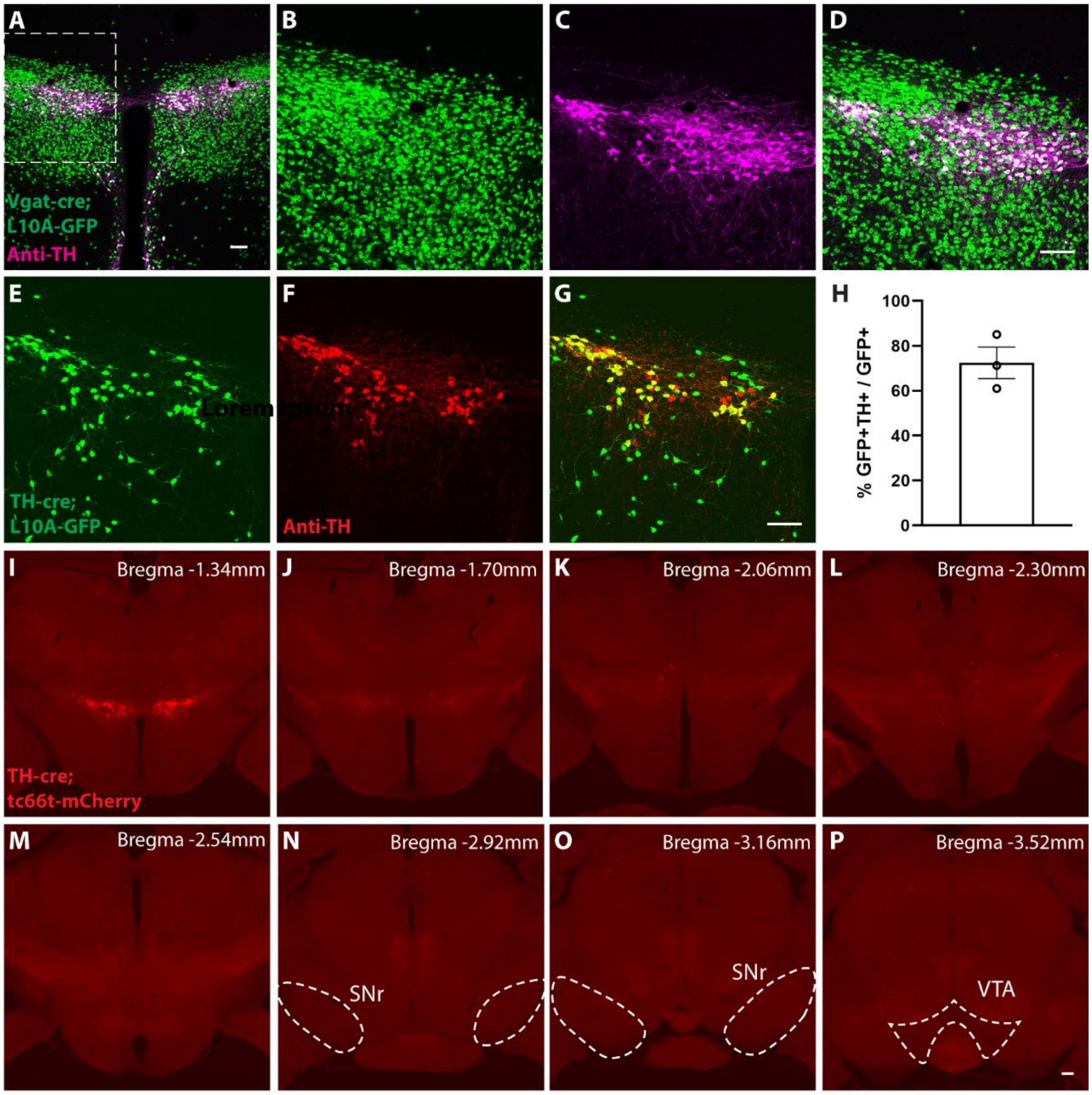

Fig S1. Validation of TH-cre mouse line and specificity of AAV infection of ZI^{TH} neurons.

A-D) ZI^{TH} neurons colocalize with Vgat, a GABAergic neuron marker;

E-H) Validation of TH-cre mouse line (N= 3 mice) (scale bar, 100 μm);

I-P) Confocal images from anterior to posterior to show the specificity of AAV infection. No transduction exists in the SNr or VTA (scale bar, 200 μm).

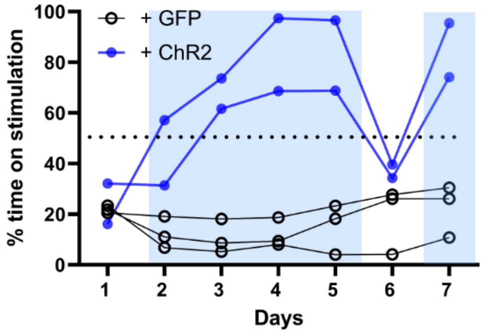

Fig S2. Acute inhibition of ZITH neurons does not induce conditioned place preference
Daily training increases time spent on the stimulated side in TH-ChR2 mice, but the preference doesn't convert to contextual memory. Laser stimulation resumes the preference on day 7 (control: N=3 mice, ChR2: N=2 mice)

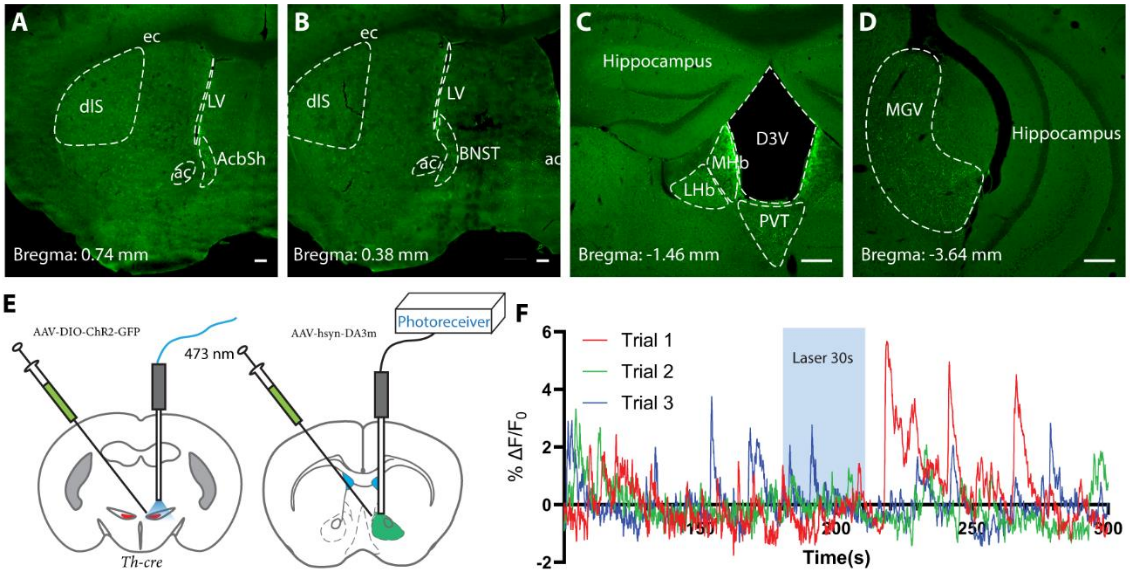

Fig S3. ZITH neurons does not project to dlS or hippocampus, or elicit reliable DA release in the NAc.

A-D) ZITH neurons do not project to the dorsolateral striatum (dlS) or hippocampus. Abbrev: ac, anterior commissure; LV, lateral ventricle; ec, external capsule; AcbSh, nucleus accumbens, shell; BNST, bed nucleus of stria terminalis; D3V, the third ventricle, dorsal part; MHb, medial habenula nucleus; LHb, lateral habenula nucleus; PV, paraventricular thalamic nucleus; MGV, medial geniculate nucleus, ventral part. scale bar, 200µm.

E) Schematic diagram of strategy to measure DA release in the NAc with ZITH neurons activation;

F) DA signaling before, during, and after optogenetic activation of ZITH neurons (n=3 trials from one mouse).

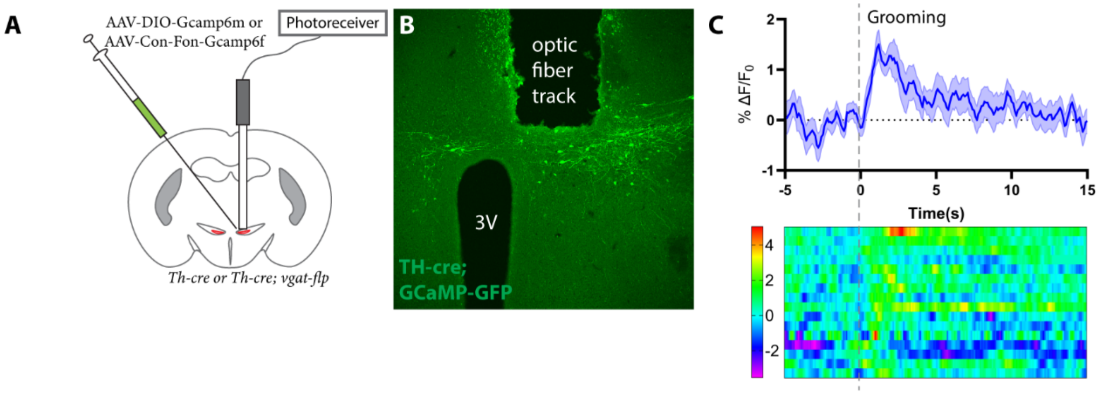

Fig S4. ZITH neurons showed increased neuronal activity during spontaneous grooming.
A) Experimental setup of recording calcium signalings from ZITH neurons;
B) a representative image of GCaMP expression in the ZI and the location of implanted optic fiber;
C) ZITH neurons showed increased neuronal activity during spontaneous grooming (n = 16 trials from N = 2 mice).

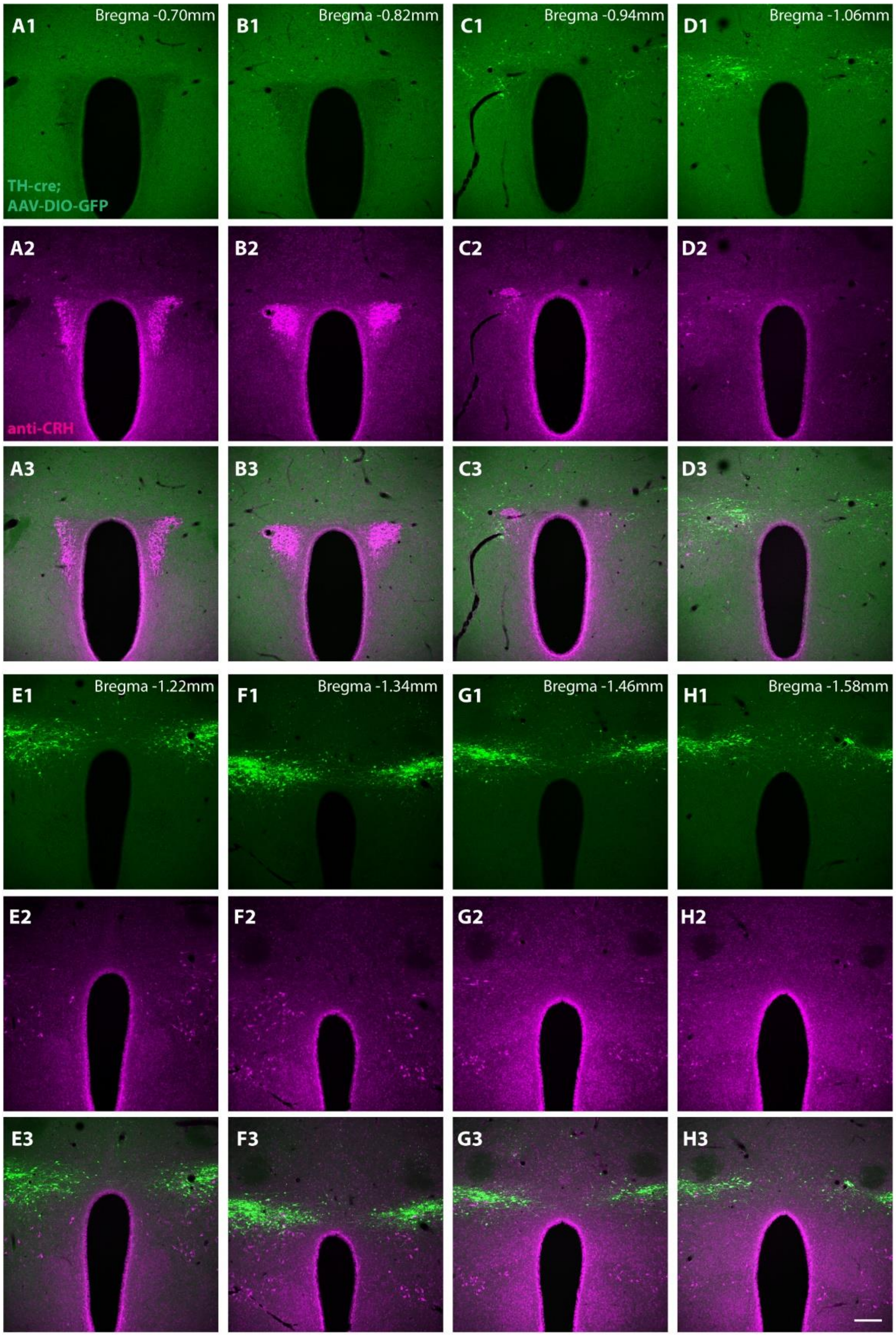

Fig S5. Confocal images from anterior to posterior show that PVH CRH neurons (magenta) do not co-localize with ZI^{TH} neurons (green) (scale bar = 200 μm).

Table S1. Downstream targets of ZI^{TH} neurons (anterior to posterior).

Brain Division	Abbreviation	Region name
Striatum	ACB	Nucleus accumbens
Striatum	LSv	Lateral septal nucleus, ventral part
Pallidum	BST	Bed nuclei of the stria terminalis
Hypothalamus	MPO	Medial preoptic area
Hypothalamus	PVH	Paraventricular hypothalamic nucleus
Hypothalamus	AHN	Anterior hypothalamic nucleus
Hypothalamus	LHA	Lateral hypothalamic area
Hypothalamus	DMH	Dorsomedial nucleus of the hypothalamus
Hypothalamus	ARH	Arcuate hypothalamic nucleus
Hypothalamus	ZI	Zona incerta
Thalamus	PVT	Paraventricular nucleus of the thalamus
Thalamus	LHb	lateral habenular nucleus
Hypothalamus	PH	Posterior hypothalamic nucleus
Midbrain	PAG	Periaqueductal gray
Thalamus	MGv	Medial geniculate complex, ventral part
fiber tracts	cic	inferior colliculus commissure
Pons	LC	Locus ceruleus
Medulla	NTS	Nucleus of the solitary tract
Medulla	LRN	Lateral reticular nucleus

Table S2. Top 50 brain regions outside of ZI that send monosynaptic projections to ZI^{TH} neurons.

Brain Division	Abbreviation	Region Name
Hypothalamus	VMH	Ventromedial hypothalamic nucleus
Midbrain	PAG	Periaqueductal gray
Hypothalamus	PVH	Paraventricular hypothalamic nucleus
Hypothalamus	PH	Posterior hypothalamic nucleus
Hypothalamus	AHN	Anterior hypothalamic nucleus
Hypothalamus	LHA	Lateral hypothalamic area
Hypothalamus	DMH	Dorsomedial nucleus of the hypothalamus
Midbrain	MRN	Midbrain reticular nucleus
Striatum	LSr	Lateral septal nucleus, rostral (rostroventral) part
Hypothalamus	PVHd	Paraventricular hypothalamic nucleus, descending division
Hypothalamus	MPO	Medial preoptic area
Hypothalamus	MPN	Medial preoptic nucleus
Pallidum	BST	Bed nuclei of the stria terminalis
Thalamus	PVT	Paraventricular nucleus of the thalamus
Pons	PRNc	Pontine reticular nucleus, caudal part
Pons	PB	Parabrachial nucleus
Hypothalamus	ARH	Arcuate hypothalamic nucleus
Hypothalamus	PeF	Perifornical nucleus
Midbrain	CUN	Cuneiform nucleus
Hypothalamus	TU	Tuberal nucleus
Hypothalamus	LPO	Lateral preoptic area
Midbrain	SCdg	Superior colliculus, motor related, deep gray layer
Striatum	ACB	Nucleus accumbens
Midbrain	PPN	Pedunculopontine nucleus
Medulla	GRN	Gigantocellular reticular nucleus
Midbrain	SCiw	Superior colliculus, motor related, intermediate white layer
Pallidum	SI	Substantia innominata
Hypothalamus	PVi	Periventricular hypothalamic nucleus, intermediate part
Pons	PRNr	Pontine reticular nucleus
Hypothalamus	PMv	Ventral premammillary nucleus
Hypothalamus	PMd	Dorsal premammillary nucleus
Hypothalamus	AVPV	Anteroventral periventricular nucleus
Thalamus	VM	Ventral medial nucleus of the thalamus
Midbrain	SCig	Superior colliculus, motor related, intermediate gray layer
Midbrain	APN	Anterior pretectal nucleus
Thalamus	SPFm	Subparafascicular nucleus, magnocellular part
Hypothalamus	PSTN	Parasubthalamic nucleus
Hypothalamus	VMPO	Ventromedial preoptic nucleus
Hypothalamus	PVp	Periventricular hypothalamic nucleus, posterior part
Isocortex	RSPv5	Retrosplenial area, ventral part, layer 5
Midbrain	SCdw	Superior colliculus, motor related, deep white layer
Thalamus	PR	Perireunensis nucleus

Striatum	LSv	Lateral septal nucleus, ventral part
Midbrain	RN	Red nucleus
Pons	PCG	Pontine central gray
Hypothalamus	FF	Fields of Forel
Thalamus	PT	Parataenial nucleus
Hypothalamus	AVP	Anteroventral preoptic nucleus
Thalamus	LGv	Ventral part of the lateral geniculate complex
Midbrain	PRC	Precommissural nucleus
